# Wheat Flour Intake Promotes Weight Gain and Metabolic Changes in Mice

**DOI:** 10.1002/mnfr.70394

**Published:** 2026-01-22

**Authors:** Shigenobu Matsumura, Miona Marutani, Eri Nousou, Nagisa Murakami, Saki Mizobata, Miyu Fujisawa, Mizuki Fujiwara, Nanase Iki, Soyoka Horie, Yuka Yamato, Azumi Yamamoto, Mina Fujitani, Teppei Fujikawa, Chinami Ishibashi, Shigeo Takenaka

**Affiliations:** ^1^ Department of Nutrition Osaka Metropolitan University Osaka Japan; ^2^ Center For Hypothalamic Research, Department of Internal Medicine UT Southwestern Medical Center Dallas Texas USA

**Keywords:** bread, metabolomics, obesity, wheat flour

## Abstract

This study examined the metabolic effects of wheat flour intake on body weight regulation in mice. Male and female C57BL/6 mice were given free access to standard chow and wheat‐based foods, including bread and baked wheat flour, and food preference, energy expenditure, hepatic gene expression, and blood metabolite profiles were analyzed. Mice showed a strong preference for wheat‐based foods, leading to significant body weight gain despite comparable caloric intake. Wheat flour consumption was associated with reduced energy expenditure, increased adiposity, and elevated circulating insulin and leptin levels. Blood metabolomic analysis revealed increased fatty acid levels and reduced essential amino acids, suggesting enhanced lipogenesis and a potential imbalance in amino acid intake. Consistently, hepatic expression of genes involved in fatty acid synthesis and lipid transport was upregulated. Importantly, withdrawal of wheat flour rapidly attenuated body weight gain and reversed the associated metabolic alterations. These findings demonstrate that wheat flour intake promotes obesity in mice primarily by decreasing energy expenditure and altering metabolic pathways independent of excess calorie consumption, highlighting wheat flour as a dietary factor that strongly influences energy homeostasis and body weight regulation.

## Graphical abstract

When given a choice between standard chow and wheat‐based foods such as bread or baked wheat flour, mice consistently preferred the wheat products. This selective intake led to significant weight gain despite unchanged total caloric consumption. Wheat flour intake was associated with reduced energy expenditure, enhanced hepatic fatty acid synthesis, and altered blood metabolite profiles, including increased long‐chain fatty acids and decreased essential amino acids. These findings suggest that wheat flour, even without added sugars or fats, can disrupt energy balance and amino acid metabolism, contributing to obesity development.

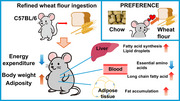

**Abbreviations**
Acaca: Acetyl‐CoA Carboxylase Alpha
Apob: Apolipoprotein B
BAT: Brown Adipose Tissue
BCAA: Branched‐Chain Amino Acids
BODIPY: Boron‐Dipyrromethene
BSTFA: N,O‐Bis(trimethylsilyl)trifluoroacetamide
DAPI: 4′,6‐Diamidino‐2‐Phenylindole
Elovl6: Elongation of Very Long Chain Fatty Acids 6
Fasn: Fatty Acid Synthase
HFD: High‐Fat Diet
Mttp: Microsomal Triglyceride Transfer Protein
PCA: Principal Component Analysis
RER: Respiratory Exchange Ratio
Rplp0: Ribosomal Protein Lateral Stalk Subunit P0
WAT: White Adipose Tissue

## 1 Introduction

Obesity is a major risk factor for various metabolic disorders including diabetes, dyslipidemia, and cardiovascular diseases [1, 2]. The global prevalence of obesity continues to increase, and an increasing number of individuals are affected by obesity‐related health conditions [3, 4]. Consequently, the prevention of obesity has become a critical focus of public health strategies. Although a genetic predisposition contributes to obesity, lifestyle factors, particularly unhealthy dietary habits, play a central role. Dietary fat has been identified as a key contributor to obesity [5, 6, 7]. High‐fat foods are not only energy‐dense, but also highly palatable, which increases the likelihood of excessive energy intake [8, 9, 10]. Compared to carbohydrates and proteins, fats are more energy‐dense and palatable, often leading to an energy intake that exceeds expenditure, thereby promoting fat accumulation and weight gain. Thus, the high palatability and energy density of fats are considered the major drivers of overeating and obesity development.

Conversely, the contribution of excessive carbohydrate intake to obesity is often underestimated, despite substantial evidence of its role [11, 12, 13]. Overconsumption of carbohydrates has been implicated in the onset of metabolic disorders, such as diabetes and dyslipidemia [14, 15, 16, 17, 18, 19]. Carbohydrate‐rich foods, particularly those made from wheat such as bread, cookies, cakes, and noodles, are widely consumed because of their palatability. However, the appealing taste of wheat‐based products may promote overconsumption, leading to increased energy intake, fat accumulation, and weight gain [20, 21]. Although there is widespread perception that wheat‐based foods promote weight gain [22, 23, 24], the mechanisms underlying excessive wheat consumption and its association with obesity remain poorly understood.

To address this gap, we investigated the effects of bread and wheat flour consumption on body weight and energy metabolism in mice. Additionally, we examined changes in liver gene expression and blood metabolite profiles to better understand the influence of wheat flour intake on energy homeostasis.

## 2 Materials and Methods

### 2.1 Animals

Animal experiments were approved by the Osaka Metropolitan University Animal Care and Use Committee (permission number: 24–117) and conducted in accordance with institutional guidelines and the NIH Guide for the Care and Use of Laboratory Animals [25]. Experimental procedures were designed to minimize animal use and suffering.

Mice were housed in groups (4–6 per cage) under controlled temperature (23°C ± 2°C) and a 12‐h light/dark cycle. At the end of experiments, animals were euthanized by isoflurane anesthesia or pentobarbital administration (150 mg/kg), followed by cervical dislocation. Detailed procedures were performed as previously described [26].

### 2.2 Preparation of Test Sample

Bread: Bread was prepared by a commercially available bread baker (Siroka, Tokyo, Japan) according to the manufacturer's instructions. The ingredients used were 320 g of high‐gluten wheat flour (Nisshin Seifun Welna Inc., Tokyo, Japan), 3 g of dry yeast (Nisshin Seifun Welna), and 230 mL of water.

Wheat flour and rice flour: The starch contained in wheat flour and rice flour must undergo gelatinization to be easily digestible and suitable for human consumption. Therefore, instead of providing wheat flour or rice flour directly to the mice, we induced starch gelatinization by mixing wheat flour or rice flour with water and baking it, similar to the process of making bread. This method allows starch to gelatinize, making it more suitable for consumption by mice.

A 2:1 ratio of high‐gluten wheat flour (Nisshin Seifun, Tokyo, Japan) and tap water was thoroughly mixed and covered with plastic wrap for 2 h. Regarding rice flour, a 5:4 ratio of rice flour (NIPPUN, Tokyo, Japan) and tap water was mixed. The dough was then rolled evenly to a thickness of approximately 3 mm. It was then baked in an oven (Sharp, Osaka, Japan) at 180°C for 10 min. After baking, the dough was torn into small pieces.

### 2.3 Voluntary Ingestion of Bread, Wheat Flour, and Rice Flour

The composition of all diets used in this experiment is shown in Table 1. Four‐week‐old male and female C57BL/6N mice were obtained from Japan SLC (Hamamatsu, Japan). Commercial standard normal chow (Chow) (MF; Oriental Yeast, Tokyo, Japan) and water were provided ad libitum. The caloric ratios of protein, fat, and carbohydrates in the Chow were 31.7%, 11.9%, and 56.4%, respectively. The mice were maintained for two weeks after their arrival to acclimatize them to their surroundings before testing. Voluntary ingestion of high fat diet (HFD), bread, wheat flour, or rice flour was initiated at six weeks of age.

**TABLE 1:** Diet composition.

	Protein (kcal%)	Carbohydrate (kcal%)	Fat (kcal%)
Commercial standard normal chow (MF: Oriental yeast)	31.7	56.4	11.9
High fat diet (HFD60: Oriental yeast)	18.1	19.9	62
Wheat flour (Nisshin Seifun Welna)	14.4	81.4	4.1
Rice flour (Nippn Corporation)	6.9	86.7	2.2

### 2.4 Wheat Flour Consumption Cessation Experiment

Mice (*n =* 21) were given Chow and wheat flour for approximately five weeks. Subsequently, the mice were divided into two groups for an additional five weeks: one group continued to receive Chow and wheat flour (wheat flour; *n =* 11), whereas the other group ceased wheat flour intake and was given Chow only (wheat cessation; *n =* 10). The intakes of Chow and wheat flour were measured separately over time.

### 2.5 Wheat Flour Consumption With HFD Intake

The animals were randomly assigned to two groups: one group was fed a HFD (HFD60: Oriental yeast, Tokyo, Japan) and chow (HFD + Chow; *n =* 7), and the other group received HFD and wheat flour (HFD + wheat flour; *n =* 6). The respective diets were administered for eight weeks. Body weights were recorded weekly. To allow ad libitum access to food, all food items were made available to the mice without restriction.

### 2.6 Respiratory Gas Analysis

Energy metabolism was assessed by indirect calorimetry using an open‐circuit metabolic gas analysis system (Arco2000, ArcoSystem, Japan), as previously reported [27]. Mice were individually housed in metabolic chambers, and oxygen consumption, carbon dioxide production, and locomotor activity were continuously monitored.

### 2.7 Tissue Collection

Liver was collected during the light phase and stored at −80°C until analysis, as previously described [27]. All the procedures were performed on ice to prevent RNA and protein degradation.

### 2.8 Blood Analysis

Blood samples were collected from mice with free access to food during the light period (3–6 h after lights on). Trunk blood was obtained, and serum was prepared by centrifugation at low temperature. Serum glucose, non‐esterified fatty acids, and triglyceride concentrations were determined using enzymatic assay kits (Wako Pure Chemical Industries, Osaka, Japan). Circulating insulin and leptin levels were measured using a mouse insulin ELISA kit (RRID:AB_2783837; Mercodia, Uppsala, Sweden) and a leptin ELISA kit (RRID:AB_2888686; BioVendor Laboratory Medicine, Brno, Czech Republic), respectively, according to the manufacturers’ instructions.

For serum metabolite extraction, 10 µL of serum was combined with 900 µL of an ethanol–water mixture (7:3, v/v) containing 10 µL of 2‐isopropylmalic acid solution (1 mg/mL in distilled water; Sigma‐Aldrich) as an internal standard [27]. After vortex mixing, the samples were centrifuged at 15,000 × g for 5 min at 4°C. An aliquot (850 µL) of the resulting supernatant was transferred to a clean 1.5‐mL tube and evaporated under a nitrogen stream, followed by overnight freeze‐drying.

For oximation, the dried extracts were reacted with 80 µL of methoxyamine hydrochloride in pyridine (20 mg/mL; Sigma‐Aldrich) at 30°C for 90 min with shaking at 1200 rpm. Subsequently, trimethylsilylation was performed by adding 40 µL of N,O‐bis (trimethylsilyl) trifluoroacetamide (BSTFA; Wako) and incubating the samples at 90°C for 30 min. The derivatized samples were then subjected to GC‐MS analysis.

GC‐MS measurements were carried out using a GCMS‐TQ8040 NX system (Shimadzu Co., Kyoto, Japan) equipped with a DB‐5 capillary column (30 m × 0.25 mm i.d., 1.00 µm film thickness; Agilent). The oven temperature was programmed from 100°C to 320°C at a rate of 10°C/min, with a total run time of 37 min. Helium was used as the carrier gas at a constant linear velocity of 39.0 cm/s, and the injector temperature was set at 280°C. Samples (1 µL) were introduced in splitless mode. Mass spectrometry was performed with an electron ionization voltage of 70 eV and an ion source temperature of 200°C.

Metabolite signals were identified and quantified using the Smart Metabolites Database (Shimadzu Co.), which incorporates multiple reaction monitoring (MRM) parameters and retention index information corresponding to the GC analytical conditions.

### 2.9 Quantitative PCR Analysis

Total RNA was isolated from whole tissues using Sepasol‐RNA I Super G (Nacalai Tesque, Kyoto, Japan) and subsequently reverse‐transcribed into complementary DNA using ReverTra Ace qPCR RT Master Mix (TOYOBO, Osaka, Japan). Quantitative PCR was performed on a LightCycler 480 system with 2× SYBR Green mix (Roche, Indianapolis, IA, USA). Relative gene expression levels were calculated using ribosomal protein lateral stalk subunit P0 (*Rplp0*) as an internal reference gene [26].

The following primer sequences were used for the PCR:

*Rplp0*, F‐5′‐AGATTCGGGATATGCTGTTGGC‐3′ and R‐5′‐TCGGGTCCTAGACCAGTGTTC‐3′; *Acaca*, F‐5′‐GATGAACCATCTCCGTTGGC‐3′ and R‐5′‐GACCCAATTATGAATCGGGAGTG‐3′; *Fasn*, F‐5′‐CACAGCATTCAGTCCTATCCACAGA‐3′ and R‐5′‐CACAGCCAACCAGATGCTTCA‐3′; *Elovl6*, F‐5′‐GAAAAGCAGTTCAACGAGAACG‐3′ and R‐5′‐AGATGCCGACCGACCACCAAAGATA‐3′; *Apob*, F‐5′‐ AAGCACCTCCGAAAGTACGTG ‐3′ and R‐5′‐ CTCCAGCTCTACCTTACAGTTGA ‐3′; *Mttp*, F‐5′‐CTCTTGGCAGTGCTTTTTCTCT‐3′ and R‐5′‐GAGCTTGTATAGCCGCTCATT‐3′.

### 2.10 Histology

Collected liver was fixed with 4% paraformaldehyde and transferred into 30% sucrose in PBS at 4°C. Samples were sliced into 15‐µm‐thick sections and collected on glass slides. Sections were stained for 60 min in BODIPY dye solution (Thermo Fisher Scientific, MA, USA) followed by counterstaining with DAPI for nuclear staining. Microscopic examination was performed using a confocal laser‐scanning microscope (FV1000; Olympus, Tokyo, Japan).

### 2.11 Statistical Analysis

All data are expressed as mean ± SEM. Statistical analyses were performed using GraphPad Prism software (version 10.0; GraphPad Software Inc., San Diego, CA, USA). Comparisons between two groups were carried out using Student's *t*‐test. For analyses of body weight changes, tissue weights, gene expression, and food intake, one‐way or two‐way repeated‐measures ANOVA was applied as appropriate. Post hoc multiple comparisons were conducted using Bonferroni's or Dunnett's tests when significant effects were detected. Statistical significance was defined as *p* < 0.05.

## 3 Results

### 3.1 Effect of Feeding Bread With Chow on Body Weight Gain

**FIGURE 1:**
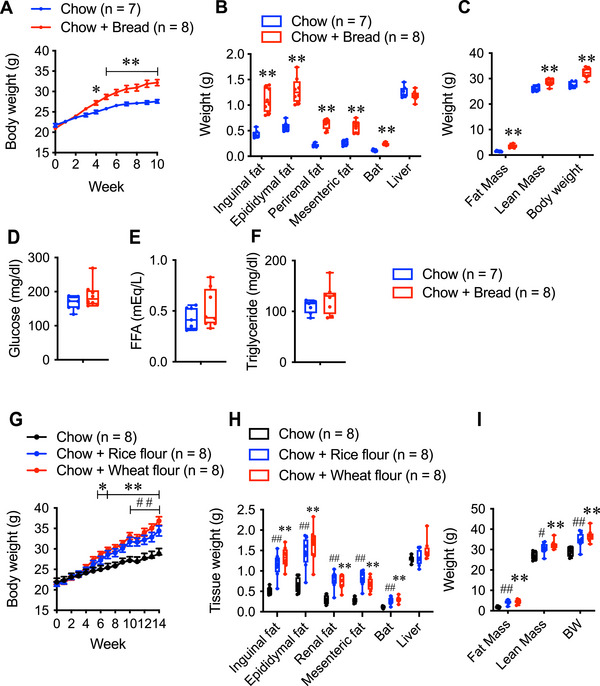
(A) Body weight change, (B) tissue weight, and (C) fat and lean mass in male mice fed chow (Chow; *n =* 7) and mice fed chow and bread (Chow + Bread; *n =* 8). (D) Levels of serum glucose, (E) free fatty acids (FFAs), (F) triglycerides in Chow and Chow + Bread mice after 10 weeks of the experiment. (G) Body weight change, (H) tissue weight, and (I) fat and lean mass in male mice fed chow (Chow; *n =* 8), mice fed chow and rice flour (Chow + Rice flour; *n =* 8), and mice fed chow and wheat flour (Chow + Wheat flour; *n =* 8). Data are presented as mean ± SEM. Statistical significance was determined using an unpaired *t*‐test (B and C), one‐way ANOVA followed by Dunnett's multiple comparisons post hoc test (H and I), or two‐way repeated‐measures ANOVA followed by Bonferroni's multiple comparisons post hoc test (A and G). **P and ## *P* indicate *p* < 0.01; * *P* and # *P* indicate *p* < 0.05. * *P* denotes a significant difference between Chow and Wheat flour, whereas #*P* denotes a significant difference between Chow and Rice flour. In the box and whisker plots, the median value is indicated by the horizontal dividing line, with the top and bottom of the box indicating the 75th and 25th percentiles, respectively, and the whiskers indicating the maximum and minimum points.

Under bread‐feeding conditions, the mice showed significant differences in body weight gain after the fourth week of the experimental period (Figure 1A). At the end of the experiment, a significant increase in white adipose tissue (WAT: Inguinal fat, epididymal fat, perirenal fat, and mesenteric fat) and interscapular brown adipose tissue (BAT) size was observed in mice fed bread, and the liver size was comparable to that of control mice (Figure 1B). Both fat mass and lean mass were significantly higher in the bread‐fed group (Figure 1C). No significant differences were observed in blood glucose, triglyceride, or free fatty acid levels among the groups (Figure 1D–F).

To examine the effects of rice flour intake on body weight, we provided mice with either rice flour or wheat flour and compared their body weight gain to that of chow‐fed controls. Both the wheat flour‐fed and rice flour‐fed groups showed significant increases in body weight compared to the chow group by six weeks after the start of feeding (Figure 1G). At the end of the experiment, white and brown adipose tissue weights were significantly increased in both groups, while liver weight remained unchanged (Figure 1H). Both fat mass and lean mass were higher in the rice flour‐ and wheat flour‐fed mice compared to controls (Figure 1I).

### 3.2 Effect of Feeding Wheat Flour on Body Weight Gain in Male Mice

**FIGURE 2:**
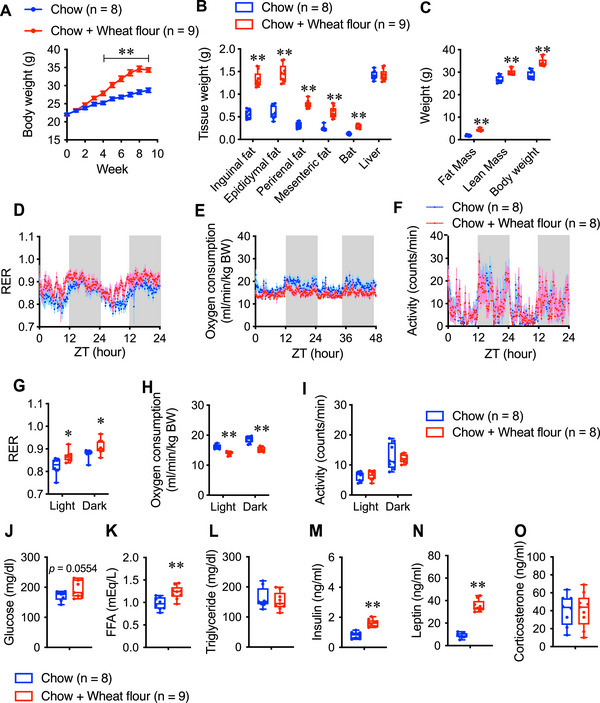
(A) Body weight change, (B) tissue weight, and (C) fat and lean mass in male mice fed chow (Chow; *n =* 8) and male mice fed chow and wheat flour (Chow + Wheat flour; *n =* 9). (D) Respiratory exchange ratio (RER), (E) oxygen consumption, and (F) spontaneous motor activity. (G) Average RER, (H) average oxygen consumption, and (I) average motor activity for 12 h (light and dark phases). (J) Levels of serum glucose, (K) free fatty acids (FFAs), (L) triglycerides, (M) insulin, (N) leptin, and (O) corticosterone in Chow and Chow + Wheat flour mice after nine weeks of the experiment. Data are represented as the mean ± SEM. **p < 0.01 and *p < 0.05 via unpaired *t*‐test (B, C, and G‐O) or two‐way repeated‐measures ANOVA, followed by Bonferroni's multiple comparisons post‐hoc test (A). In the box and whisker plots, the median value is indicated by the horizontal dividing line, with the top and bottom of the box indicating the 75th and 25th percentiles, respectively, and the whiskers indicating the maximum and minimum points.

We next investigated whether wheat flour and bread could contribute to an increase in body weight gain. Significant weight gain was observed in the fourth week of wheat flour supplementation (Figure 2A). At the end of the experiment, WAT and BAT weights were significantly increased in wheat flour‐fed mice (Figure 2B), along with elevated fat and lean mass (Figure 2C).

*Respiratory gas analysis*: Wheat flour feeding significantly increased the respiratory exchange ratio (RER) and decreased oxygen consumption during both the light and dark phases (Figure 2D,E,G,H), while motor activity remained comparable between groups in both phases (Figure 2F,I).

*Blood analysis*: After nine weeks, wheat flour‐fed mice showed significantly elevated levels of free fatty acids, insulin, and leptin (Figure 2K,M,N), while blood glucose, triglyceride, and corticosterone remained comparable to those of controls (Figure 2J,L,O).

### 3.3 Effect of Feeding Wheat Flour on Body Weight Gain in Female Mice

Female C57BL/6 mice are resistant to diet‐induced obesity, likely due to estrogenic effects [28, 29, 30]. To assess the impact of wheat flour on females, we monitored body weight changes over time.

**FIGURE 3:**
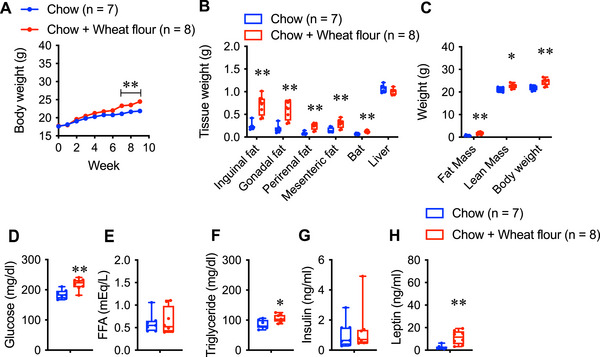
(A) Body weight change, (B) tissue weight, and (C) fat and lean mass in female mice fed chow (Chow; *n =* 7) and mice fed chow and wheat flour (Chow + Wheat flour; *n =* 8). (D) Levels of serum glucose, (E) free fatty acids (FFAs), (F) triglycerides (TGs), (G) insulin and (H) leptin in both groups after nine weeks of wheat flour intake. Data are represented as the mean ± SEM. **p < 0.01 and *p < 0.05 via unpaired *t*‐test (B‐H) or two‐way repeated‐measures ANOVA, followed by Bonferroni's multiple comparisons post‐hoc test (A). In the box and whisker plots, the median value is indicated by the horizontal dividing line, with the top and bottom of the box indicating the 75th and 25th percentiles, respectively, and the whiskers indicating the maximum and minimum points.

In male mice, weight gain began in the fourth week of wheat flour consumption. In contrast, female mice showed no significant weight change until the sixth week, with a significant increase observed from the seventh week onward (Figure 3A). WAT and BAT weights were significantly higher in wheat flour‐fed females, whereas the liver weight remained unchanged (Figure 3B). Both fat and lean mass increased (Figure 3C).

Blood glucose, triglyceride, and leptin levels were significantly elevated in wheat flour‐fed females, whereas free fatty acid and insulin levels showed no significant differences (Figure 3D–H).

### 3.4 Effect of Voluntary Ingestion of Wheat Flour on Blood Metabolite

**FIGURE 4:**
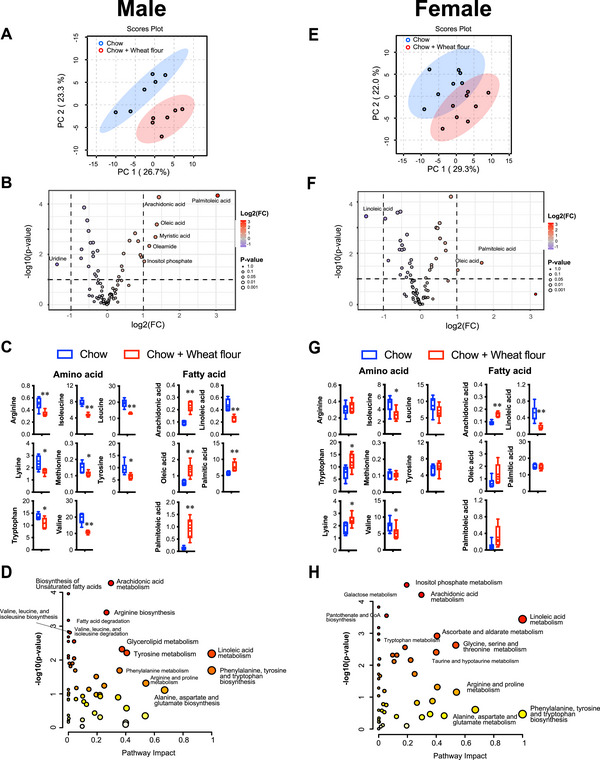
Targeted metabolomics analysis of the male mice blood using Metaboanalyst ver.6.0. (A, E) Principal component analysis (PCA) score plots of serum metabolites in male (A) and female (E) mice. (B, F) Volcano plots showing differential metabolite profiles in male (B) and female (F) mice. (C, G) Comparison of essential amino acid levels in male (C) and female (G) mice. (D, H) Summary of metabolic pathways associated with wheat flour intake, based on pathway enrichment analysis in male (D) and female (H) mice. The color of the circles from white to yellow to red denotes incremental fold change (‐log(p)). The size of the circles from small to large indicates an increment of the impact of pathway. **p < 0.01 and *p < 0.05 by unpaired *t*‐test (C and G). In the box and whisker plots, the median value is indicated by the horizontal dividing line, with the top and bottom of the box indicating the 75th and 25th percentiles, respectively, and the whiskers indicating the maximum and minimum points.

To investigate the effects of wheat flour intake on metabolism, we analyzed blood metabolites using gas chromatography‐mass spectrometry (GC/MS). GC/MS analysis identified 86 metabolites in serum samples. These metabolites were further analyzed using MetaboAnalyst version 6.0. The PCA score (Figure 4A,E) and volcano plots (Figure 4B,F) revealed a clear separation between the two groups based on their blood metabolite profiles.

In male mice that consumed wheat flour, levels of all eight essential amino acids were lower than in controls (Figure 4C). Levels of unsaturated fatty acids (oleic acid, palmitoleic acid) and the saturated fatty acid palmitic acid were elevated, while the n‐6 fatty acid arachidonic acid increased and its precursor linoleic acid decreased.

Wheat flour intake influenced several metabolic pathways, including unsaturated fatty acid biosynthesis, arachidonic acid metabolism, arginine biosynthesis, fatty acid degradation, branched‐chain amino acid (BCAA) biosynthesis and degradation (valine, leucine, and isoleucine), glycerolipid metabolism, tyrosine metabolism, and linoleic acid metabolism (Figure 4D).

In contrast, in female mice that consumed wheat flour, only isoleucine and valine levels were decreased (Figure 4G). Increased lysine and tryptophan levels were also observed. Similar to male mice, arachidonic acid levels were elevated, whereas linoleic acid levels were reduced.

### 3.5 Effect of Wheat Flour Intake on Liver Gene Expression and Histology

**FIGURE 5:**
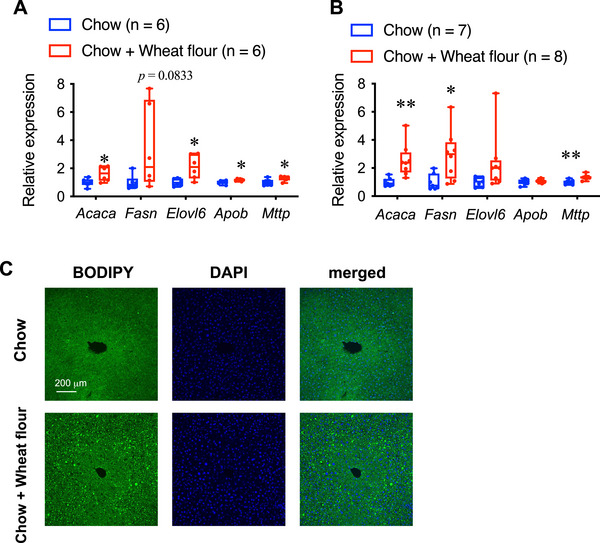
(A, B) mRNA expression levels in the liver of male (A) and female (B) mice fed a standard chow diet (Chow) and male mice fed chow and wheat flour (Chow + Wheat flour). **p < 0.01 and *p < 0.05 by unpaired *t*‐test. In the box and whisker plots, the median value is indicated by the horizontal dividing line, with the top and bottom of the box indicating the 75th and 25th percentiles, respectively, and the whiskers indicating the maximum and minimum points. (C) Liver histology. Lipid droplets and nuclei were stained by BODIPY (green) and DAPI (blue).

To investigate the effects of flour intake on hepatic lipid metabolism, we measured the mRNA expression of enzymes in fatty acid synthesis. In male mice fed wheat flour, the hepatic mRNA levels of *Acaca*, *Fas*, and *Elovl6* were elevated compared with those in the control group (Figure 5A).

We also examined triglyceride transport and found that the mRNA expression levels of *Mttp* and *Apob*, key components of lipoproteins, increased in the wheat flour‐fed group.

In female mice fed wheat flour, the hepatic mRNA levels of *Acaca*, *Fas*, and *Mttp* were elevated compared with those in the control group (Figure 5B).

Given that wheat consumption was found to promote hepatic lipogenesis, we subsequently conducted a histological analysis of the livers of male mice that consumed wheat flour for 14 weeks. Hepatic lipid droplets were scarcely observed in mice fed the standard diet (Figure 5C). In contrast, numerous lipid droplets were detected in the livers of the mice that consumed wheat flour.

### 3.6 Effect of Cessation of Wheat Flour Intake on Body Weight Gain

Continuous consumption of wheat flour in mice leads to significant weight gain and reduced energy expenditure, accompanied by changes in energy metabolism‐related gene expression across multiple organs. To determine whether these effects persist after wheat flour withdrawal, we investigated the impact of discontinuing wheat flour intake following prolonged consumption.

**FIGURE 6:**
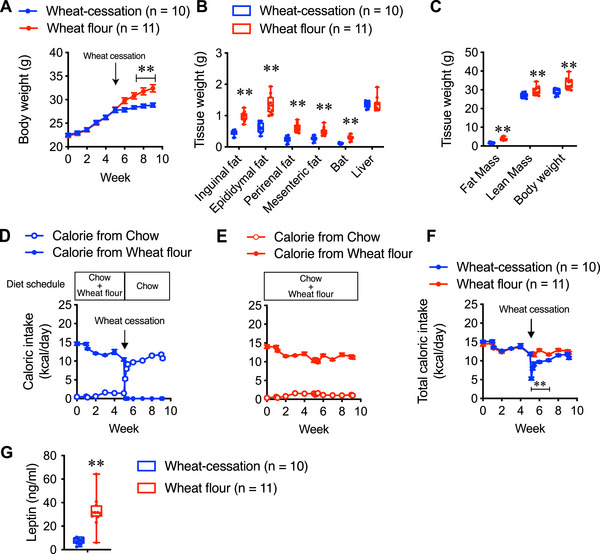
(A) Body weight change before and after a cessation of wheat flour feeding. All mice were fed both chow and wheat flour until fifth week. In Wheat‐cessation group, after fifth week, wheat flour feeding was stopped, and mice were fed only chow. In Wheat flour group, mice were fed both chow and wheat flour throughout the entire experimental period. The black arrow indicates the time point when wheat flour feeding was stopped. (B) Tissue weight and (C) fat mass and lean mass at the end of experiment. (D) Caloric intake from chow and wheat flour in Wheat‐cessation group, (E) Wheat flour group, and (F) total caloric intake in both groups. (G)Levels of leptin at the end of the experiments. Data are represented as the mean ± SEM. **p < 0.01 and *p < 0.05 via unpaired *t*‐test (B, C, and G) or two‐way repeated‐measures ANOVA, followed by Bonferroni's multiple comparisons post‐hoc test (A and F). In the box and whisker plots, the median value is indicated by the horizontal dividing line, with the top and bottom of the box indicating the 75th and 25th percentiles, respectively, and the whiskers indicating the maximum and minimum points.

During the initial five weeks, all mice were fed both chow and wheat flour. Based on weight gain and intake levels, the mice were then divided into two groups: one group continued to receive both chow and wheat flour, while the other group ceased wheat flour intake and received only chow (wheat cessation group). Within one week after wheat cessation, body weight gain ceased and subsequently stabilized in the wheat cessation group, whereas the group that continued to consume wheat flour showed progressive weight gain (Figure 6A).

At the end of the experiment, the wheat‐cessation group had significantly lower WAT and BAT than the continuous wheat consumption group, whereas the liver weight remained unchanged (Figure 6B,C).

Caloric intake analysis revealed that during the wheat feeding phase, mice in the wheat cessation group derived most of their calories from wheat flour with minimal chow intake (Figure 6D). Immediately after wheat cessation, chow intake in the wheat‐cessation group increased slightly but not substantially, leading to a marked reduction in total caloric intake compared with both the pre‐cessation period and the wheat flour‐fed group (Figure 6D,F). Over the following three days, chow intake of the wheat‐cessation group rose sharply and then continued to increase gradually. After three weeks of wheat cessation, no significant difference in total caloric intake was observed between the wheat‐cessation and wheat flour‐fed groups. In contrast, mice in the continuous wheat consumption group consistently consumed wheat flour as their primary energy source with little chow intake throughout the 10‐week period (Figure 6E).

### 3.7 Effect of Simultaneous Intake of Wheat Flour and High‐fat Diet on Body Weight Gain

As shown in Figures 6D and 6E, the mice preferred wheat flour to standard chow. To further explore this preference in the context of a highly palatable diet, we conducted an experiment in which mice were given simultaneous access to a high‐fat diet (HFD) and either standard chow or wheat flour.

**FIGURE 7:**
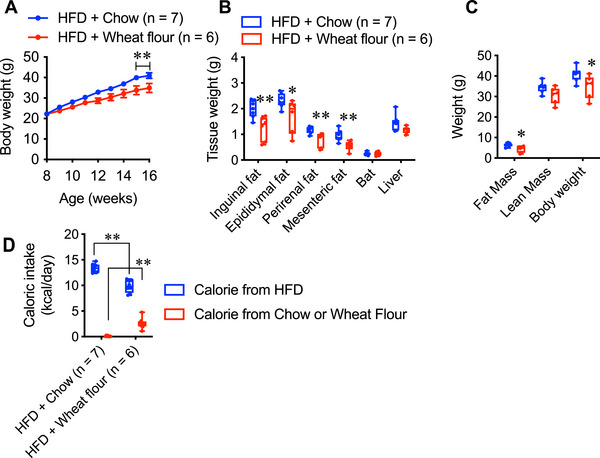
(A) Body weight change, (B) tissue weight, and (C) fat and lean mass in mice fed a high‐fat diet and chow (HFD + Chow; *n =* 7), and mice fed a high‐fat diet and wheat flour (HFD + Wheat flour; *n =* 6). (D) Caloric intake from a high‐fat diet, chow, or wheat flour in both groups. Data are represented as the mean ± SEM. **p < 0.01 and *p < 0.05 via unpaired *t*‐test (B, C and D), or two‐way repeated‐measures ANOVA, followed by Bonferroni's multiple comparisons post‐hoc test (A). In the box and whisker plots, the median value is indicated by the horizontal dividing line, with the top and bottom of the box indicating the 75th and 25th percentiles, respectively, and the whiskers indicating the maximum and minimum points.

Mice were divided into two groups: HFD + chow and HFD + wheat flour. Body weight gain was monitored throughout the experiment. Interestingly, mice in the HFD + wheat flour group exhibited attenuated weight gain compared to those in the HFD + chow group (Figure 7A). Furthermore, the WAT weights were significantly lower in the HFD + wheat flour group (Figure 7B), whereas the interscapular BAT and liver weights remained unchanged. The fat mass was reduced in the wheat flour group, whereas the lean mass was unaffected (Figure 7C).

In the HFD + chow group, the mice consumed negligible amounts of chow and primarily ingested HFD. In contrast, mice in the HFD + wheat flour group consumed approximately 1 g of wheat flour in addition to HFD, resulting in a reduced overall HFD intake (Figure 7D). Consequently, the total caloric intake in the HFD + wheat flour group was lower than that in the HFD + Chow group.

## 4 Discussion

Carbohydrate intake is closely associated with obesity and metabolic syndromes in humans [14, 16, 19]. Among dietary carbohydrates, wheat is a major global source commonly consumed in the form of bread, pasta, and noodles. Previous studies using cafeteria‐style or Western diets in mice have associated wheat‐based products, such as cookies and biscuits, with obesity [31, 32]. However, the specific effects of bread consumption have not been directly examined. Moreover, the obesogenic effects of wheat are often attributed to co‐ingested fats and sugars, and the independent effects of wheat flour remain unclear.

In this study, we investigated whether mice, like humans, exhibit a preference for bread and whether this preference leads to overeating and obesity. To isolate the effects of wheat flour, we used a simplified bread formulation containing only wheat flour, water, and yeast and allowed mice to freely choose between bread and standard chow. The mice that consumed bread showed significant weight gain. To further eliminate the influence of yeast, baked wheat flour without yeast was tested. Similar results were observed, suggesting that wheat flour contributed to weight gain in mice.

Next, we examined the effect of rice flour, which is widely consumed around the world, on body weight gain. We found that rice flour also caused significant body weight gain. This indicates that mice prefer not only wheat flour but also rice flour. It should be noted that the chow diet used in this study contained more dietary fiber than the refined wheat or rice flour diets, which could potentially affect feeding behavior and digestive kinetics under ad libitum conditions [33, 34]. However, since total energy intake was largely comparable among the groups, the differences in body weight and energy expenditure are unlikely to be solely attributable to the fiber content of the diets. These findings suggest that refined wheat and rice flour may have direct effects on metabolic regulation independent of dietary fiber levels.

To explore the mechanisms underlying the body weight gain induced by wheat flour feeding, we assessed both energy intake and expenditure. Although total caloric intake did not differ significantly between the wheat flour‐fed and control groups (Figure 6F), energy expenditure analyses revealed notable differences. Despite similar levels of activity between the groups, energy expenditure decreased in wheat flour‐fed mice during both the light and dark phases. These findings suggest that the observed weight gain may be attributed to a reduction in energy expenditure associated with wheat flour consumption.

Blood glucose levels in the wheat flour‐fed group showed considerable inter‐individual variability and did not differ significantly from those of the control group. In contrast, wheat flour intake led to an increase in blood insulin levels, suggesting that consumption of wheat flour may contribute to obesity and insulin resistance.

Although dietary habits and stress hormones such as corticosterone are often implicated in energy balance [35, 36], our data indicate that wheat flour consumption did not alter circulating corticosterone levels. This suggests that the observed effects of wheat flour on body weight gain and energy expenditure are not mediated by corticosterone. Stress or exercise may still influence these outcomes via other mechanisms, such as increased energy expenditure or changes in substrate utilization, but the corticosterone pathway is unlikely to be involved in this model.

Given that female C57BL/6 mice are reportedly resistant to diet‐induced obesity due to the protective effects of estrogen [28, 29, 30], we also examined the impact of wheat flour intake in female mice. Consistent with previous findings, weight gain in females was attenuated compared to males, suggesting that estrogen may mitigate the obesogenic effects of wheat flour, as observed in high‐fat diet models [29]. However, wheat flour ingestion results in elevated blood glucose levels in females, suggesting a potential impairment in glucose metabolism. Despite this, the insulin levels remained unchanged, indicating a possible reduction in insulin sensitivity.

Both male and female mice fed with wheat flour exhibited significantly elevated plasma leptin levels, likely reflecting increased adiposity. Additionally, female mice had higher plasma triglyceride levels, suggesting sex‐specific differences in wheat flour digestion, absorption, and metabolism.

Increased mRNA expression of *Acaca, Fas, and Elovl6* was observed in mice fed wheat flour. This suggests that de novo fatty acid synthesis from the carbohydrates derived from wheat flour has advanced. Furthermore, the expression levels of *Mttp* and *Apob* were increased, suggesting that triglycerides synthesized in the liver are transported to the whole body as lipoproteins, causing excessive fat accumulation.

Blood metabolomics were performed to observe the metabolic changes caused by wheat flour intake. As shown in the PCA score plot, wheat flour intake caused changes in blood metabolites, and the metabolites with significant differences were mainly related to the fatty acid and amino acid metabolism pathways.

Levels of essential amino acids, including branched‐chain amino acids (BCAAs) and aromatic amino acids, were decreased in mice that consumed wheat flour. This reduction was likely due to a marked decrease in the standard diet intake driven by a preference for wheat flour, which is composed of approximately 80% carbohydrates. Wheat flour has a poor amino acid profile, particularly lacking essential amino acids, such as lysine, tryptophan, and BCAAs [13, 37, 38]. Consequently, excessive consumption of wheat may lead to reduced intake of essential amino acids, potentially resulting in a state of protein deficiency. Protein deficiency causes weight loss in mice [39, 40] and humans [41]. Furthermore, a reduced protein intake promotes weight loss in obese individuals [42].

Despite the observed reduction in essential amino acid levels following wheat flour consumption, mice exhibited significant weight gain without a reduction in lean mass, suggesting that severe protein deficiency was not induced. Instead, it is plausible that wheat consumption decreases fatty acid oxidation and enhances de novo lipogenesis from carbohydrates, thereby contributing to fat accumulation and subsequent obesity.

Wheat flour consumption did not reduce essential amino acid levels in female mice. In contrast, increases in lysine and tryptophan levels were detected, which are typically limited to wheat. Unlike male mice, female mice did not exhibit significant weight gain, suggesting that their amino acid requirements may differ. Additionally, their preferences for wheat flour may vary. These sex‐specific differences in nutrient requirements and dietary preferences may explain the observed metabolic outcomes.

As for fatty acids, palmitic acid, palmitoleic acid, and oleic acid were increased by wheat flour intake. This may be due to the excessive synthesis of fatty acids resulting from the uptake of large amounts of carbohydrates by the liver. Arachidonic acid level was also increased by wheat flour intake. However, wheat flour does not contain arachidonic acid and cannot be synthesized in the body [43, 44]. It has been reported that intestinal bacteria produce long‐chain fatty acids [45, 46]. It is possible that the intake of wheat flour changed the intestinal flora, and that fatty acids were synthesized by intestinal bacteria and absorbed into the body, which increased in the blood. To clarify whether the increased arachidonic acid in the blood originates from intestinal bacteria, further studies, such as fecal metabolome analysis of mice fed with wheat flour, are necessary.

Age and associated hormonal changes are important factors that can influence metabolic responses to diet [47]. In the present study, all experiments were initiated using mice at six weeks of age to minimize variability due to developmental stage. Because wheat consumption can induce a state of protein deficiency, it is possible that younger mice, which have higher growth demands, might respond differently. Likewise, aged mice with altered endocrine profiles could exhibit distinct metabolic outcomes. These aspects warrant further investigation in future studies.

We observed that prolonged consumption of wheat flour increased body weight and reduced energy expenditure in mice. To determine whether these effects persisted after wheat flour withdrawal, we monitored the animals following diet cessation. Immediately after wheat cessation, body weight gain stopped and remained stable. During the first three days, caloric intake transiently and dramatically decreased compared with the pre‐cessation period, even though body weight was maintained. Thereafter, total caloric intake gradually increased but remained lower than that of the continuous wheat flour group.

The initial reluctance to consume the standard chow after wheat flour cessation likely reflected a persistent preference for wheat flour, resulting in a temporary reduction in food intake despite hunger. This early response appears to involve behavioral adaptation to the change in diet, as well as potential central nervous system (CNS) mechanisms affecting feeding motivation and alertness. Notably, these changes were accompanied by a rapid attenuation of body weight gain and reduced adipose tissue accumulation, suggesting that both behavioral and CNS‐mediated responses contributed to the short‐term metabolic effects. Food intake returned to baseline within two weeks, indicating that these effects were transient and reversible.

Interestingly, after wheat flour cessation, caloric intake and body weight trajectories began to diverge. Mice that continued consuming wheat flour exhibited ongoing weight gain, whereas those that discontinued wheat flour intake showed an attenuation of weight gain. These findings suggest that wheat flour–induced obesity can be attenuated upon cessation of wheat consumption.

We observed that wheat flour feeding elevated circulating leptin levels, whereas cessation of wheat flour intake reduced them. Given that persistent hyperleptinemia is a hallmark of leptin resistance, these findings suggest that continuous wheat flour consumption may impair leptin signaling. This alteration in leptin sensitivity could partly explain the reduced energy expenditure and enhanced body weight gain observed during wheat flour feeding.

Leptin resistance is known to develop in response to chronic overnutrition or high‐fat feeding and leads to impaired appetite and energy balance regulation [48]. Therefore, it is plausible that refined wheat intake, similar to other obesogenic diets, induces leptin resistance through sustained elevation of circulating leptin levels.

## 5 Conclusion

This study demonstrated that wheat flour intake induces obesity in mice, primarily through reduced energy expenditure and enhanced hepatic fatty acid synthesis. Metabolomic analysis revealed significant alterations in blood metabolites, particularly in fatty acid and amino acid pathways. A marked decrease in essential amino acids suggests a potential protein deficiency due to the poor amino acid profile of wheat and the reduced intake of the standard diet. Despite similar caloric intake, wheat flour‐fed mice gained weight, and cessation of wheat intake rapidly reversed these effects. These findings highlight that wheat flour is a potent dietary factor that influences energy metabolism and body weight, warranting further investigation of its nutritional and metabolic consequences.

## Conflicts of Interest

The authors declare no conflicts of interest.
